# Serine-Grafted Cu_2_O Electrode Enabling Specific β‑Hydroxybutyrate Detection by Surface Sensitization-Promoted Electrolysis in Amperometry

**DOI:** 10.1021/acs.langmuir.5c00591

**Published:** 2025-05-07

**Authors:** Ting-Chi Lo, Wen-Jyun Wang, Chih-Yen Chen, Jui-Cheng Chang, Wei-Peng Li

**Affiliations:** † Department of Medicinal and Applied Chemistry, 38023 Kaohsiung Medical University, Kaohsiung 807, Taiwan; ‡ Department of Electrophysics, National Yang Ming Chiao Tung University, Hsinchu 300, Taiwan; § Department of Chemical Engineering, 34900 Chung Yuan Christian University, Taoyuan 320, Taiwan; ∥ Department of Medical Research, Kaohsiung Medical University Hospital, Kaohsiung 807, Taiwan; ⊥ Drug Development and Value Creation Research Center, 38023 Kaohsiung Medical University, Kaohsiung 807, Taiwan; # Center of Applied Nanomedicine, National Cheng Kung University, Tainan 701, Taiwan

## Abstract

As the global prevalence of diabetes continues to rise, the home health testing market has experienced rapid growth. Although blood glucose monitoring is widespread among diabetic patients, there remains a significant lack of testing methods for diabetic ketoacidosis. The present study developed a feasible electrochemical technique for ketoacid detection using serine-immobilized copper­(I) oxide nanoparticles (Cu_2_O NPs) as the primary electrode material. Given that the serine on the nanoparticle surface enables conjugation with β-hydroxybutyrate (β-HBA) through an esterification reaction between the hydroxyl group of serine and carboxylic acid of β-HBA and another intramolecular nucleophilic acyl substitution between amine and ester groups to form irreversible amide bonding, thus resulting in the β-HBA deposition on the surface of the Cu_2_O NP-coated electrode. The quantification of β-HBA can be determined through current variations in amperometry measurement. The results showed a highly linear relationship between reductive current and β-HBA concentration at 0–20 mM, with a reasonable detection limit of 0.1 mM. Moreover, a reasonable mechanism involving the NP surface covering-mediated electrolysis enhancement was proposed. The present method reveals a promising direction in developing sensors for small molecule detection with high specificity and sensitivity.

## Graphical abstract

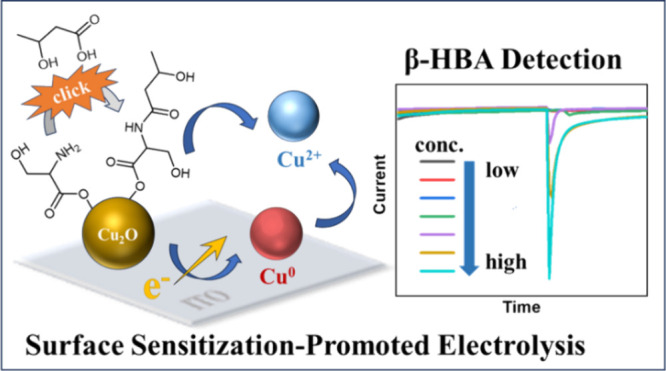

## Introduction

Diabetic ketoacidosis is one of the major complications for diabetic patients.^1^ When patients lack insulin, the activity of hormone-sensitive lipase increases, leading to the breakdown of triglycerides and the release of free fatty acids. The excessive free fatty acids overwhelm the tricarboxylic acid cycle, resulting in ketoacidosis after the metabolic conversion of acetyl-CoA into ketone bodies, such as β-hydroxybutyrate (β-HBA), acetone, and acetoacetate in the liver.^2^ Despite ongoing updates in medical management, the International Diabetes Federation estimates that the global prevalence of diabetes among people aged 20–79 is about 10.5% (536.6 million people) in 2021, and this number is expected to rise to 12.2% (783.2 million people) by 2045.^3^ Studies have shown that the current mortality rate of diabetic ketoacidosis is 0.4%, and hospitalization rates have significantly increased by 54.9%, posing a substantial medical burden.^4^

With the increasing number of patients, the diabetes device market reached a scale of 28.1 billion dollars in 2022, and it is expected to grow at an annual rate of 7.5% from 2023 to 2030, reaching 50.1 billion dollars,^5^ indicating considerable demand for diabetes monitoring devices. Several detection methods for diabetic ketoacidosis have been developed (Table S1).^6–15^ Traditional detection methods, such as enzyme-linked immunosorbent assay and high-performance liquid chromatography, although accurate and well-established, are time-consuming and costly and require specialized equipment and personnel for operation. In contrast, the Schiff base fluorescent probe offers high selectivity and rapid response, making it suitable for quick screening. However, it is susceptible to environmental light interference and suffers from photobleaching issues, necessitating stringent operational conditions.^6^ Another fluorescent chemical probe^7^ can detect β-HBA at extremely low concentrations and is applicable to various sample types, with its structural design supported by theoretical underpinnings. Nevertheless, its synthesis process is complex and prone to environmental light interference. Colorimetric methods are ideal for rapid detection due to their simplicity, ease of use, and low cost, with the color change being visually observable; however, they are subject to interference from other colored substances present in the samples, which can affect measurement accuracy.^10^ The photoelectrochemical microfluidic chip allows for the simultaneous detection of multiple diabetes biomarkers and offers high sensitivity and applicability to complex samples, although it is costly and requires specialized operation.^11^

Click chemistry is a novel synthetic technique for specifically conjugating two bioinert molecules.^16,17^ The regular functional groups in click chemistry are azides and alkynes, which can be catalyzed by copper ions to form triazole ring structures, thus efficiently creating covalent bonds between molecules. Moreover, this method enables stable and harmless reactions within biological systems; however, it also means that the click reaction has not occurred for biomolecules.^18,19^ The alternative click chemistry was further developed through well-designed specific molecule pairing, such as serine-β-HBA and glycine-acetone, thereby creating the dawn of specific small molecules captured in biosensors.^10^

Electrochemical analysis techniques involve controlling the current or voltage to trigger a target reaction, thus monitoring the meaningful electrochemical signals to achieve the detection aim. This method can obtain the required information using various electrochemical methods, such as cyclic voltammetry (CV), differential pulse voltammetry, amperometry, and square wave voltammetry, offering high flexibility and versatility.^20^ Additionally, because electrochemical instruments and equipment are easy to obtain and operate, implementing electrochemistry-based biosensors is feasible and easy to promote. Therefore, electrochemical methods have become widely used analytical tools in various fields, including environmental monitoring,^21–23^ food safety,^24–26^ and biosensors,^27,28^ and the application of electrolysis methods to achieve detection purposes is also very extensive, with anodic stripping voltammetry being the most common technique for heavy metal ion detection.^29,30^ In most cases, various electrodes integrated with enzymes or redox-active complexes were developed for the indirect detection of ketone bodies.^31,32^ A novel study indicated that using bare screen-printed carbon electrodes combined with an electrochemical probe, 2-hydrazinobenzoic acid, can detect the ketone bodies directly.^33^

This study aims to synthesize serine-modified Cu_2_O NPs (Cu_2_O-S NPs) and integrate them into an electrochemical measurement system, thus developing a new platform for the sensitive and precise detection of β-HBA. The Cu_2_O-S NP-modified electrode was fabricated and used in an electrochemical device, in which β-HBA in the buffer electrolyte can be rapidly captured through alternative click conjugation between β-HBA and serine. Moreover, the electrolysis of Cu_2_O NPs to form Cu^0^ and Cu_4_H­(PO_4_)_3_ was facilitated by β-HBA-promoted surface sensitization, thus producing the β-HBA amount-dependent reductive current and achieving direct precision evaluation of the β-HBA level in the sample (Scheme 1).

**Scheme 1:**
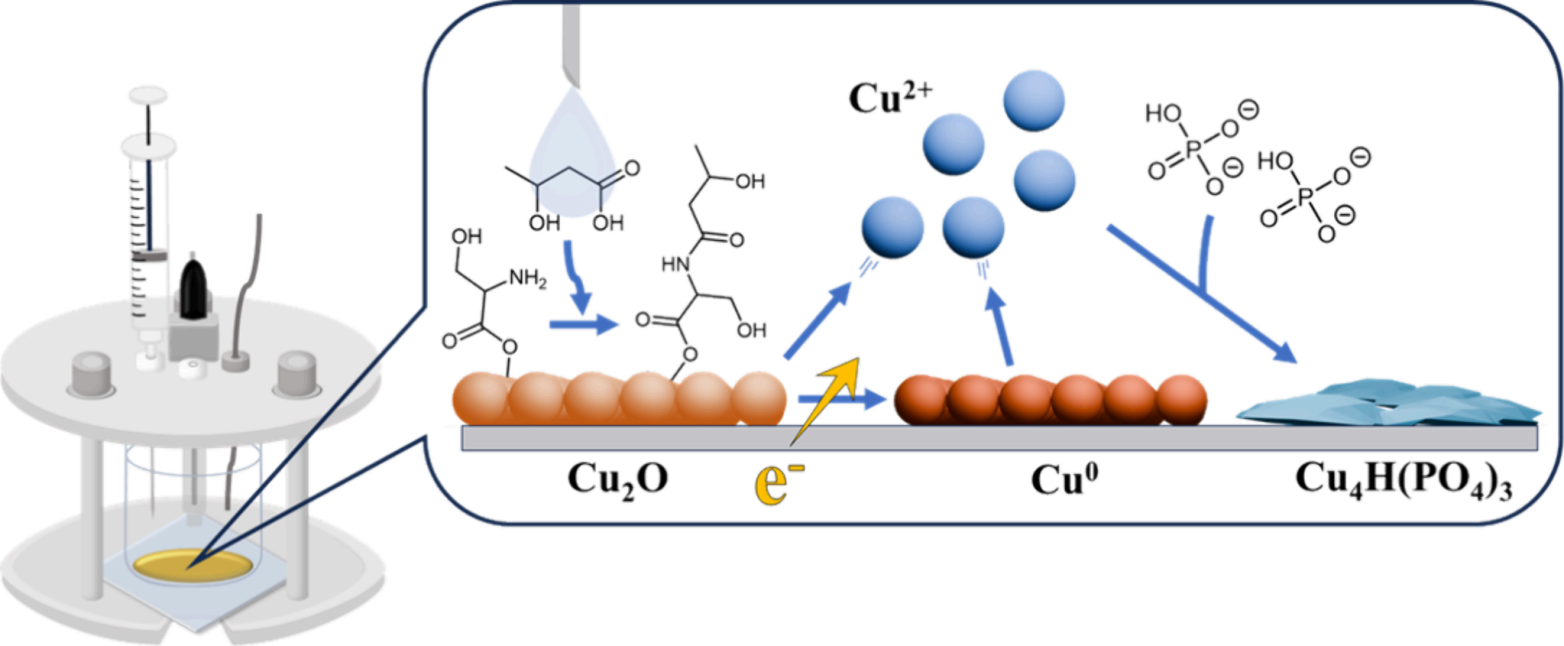
Illustration of a Feasible β-Hydroxybutyrate (β-HBA) Detection Method Involving Serine-Modified Cu_2_O Nanoparticles on the Indium Tin Oxide Electrode, Enabling the Specific Capture of β-HBA to Result in a β-HBA Concentration-Dependent Reductive Electricity Enhancement in Amperometry Analysis

## Experimental Section

### Materials

Polyvinylpyrrolidone (PVP, *M*_w_ ∼ 1,300,000 by LS), d-glucose (C_6_H_12_O_6_, ≥99.5%), hydrazine hydrate (N_2_H_4_, 50–60%), and copper­(II) nitrate hydrate [Cu­(NO_3_)_2_, 99.9%] were purchased from Sigma-Aldrich. Serine (C_3_H_7_NO_3_, 99.0%) and 4-(2-aminoethyl)­benzene-1,2-diol (C_8_H_11_NO_2_, 99.0%) were purchased from Acros Organic. Sodium chloride (NaCl, 99.0%) was purchased from Seedchem. Potassium chloride (KCl, 99.5%) and potassium phosphate monobasic (KH_2_PO_4_, ≥99.0%) were purchased from Aencore. Disodium hydrogen phosphate (Na_2_HPO_4_, 99.0%) was purchased from Showa. β-Hydroxybutyrate (C_4_H_8_O_3_, ≥80.0%) was purchased from Tokyo Chemical Industry. Ethanol (EtOH, ≥99.5%) was purchased from Echo Chemical. Water purified with a Milli-Q Synergy system was used throughout this study.

### Preparation of Cu_2_O NPs

First, 0.6 g of PVP was dissolved in 5 mL of deionized water, and 5 mL of a 30 mM Cu­(NO_3_)_2_ solution was added to the aforementioned solution while stirring continuously to mix thoroughly. Then, 8 μL of N_2_H_4_ was added to the aforementioned mixture to proceed with the reaction for 2 min. The color of the reaction solution changed from clear to yellow–brown to indicate the Cu_2_O nanoparticle formation. Afterward, the sample solution was centrifuged at 14,000 rpm for 5 min to get the Cu_2_O pellet. Then, the supernatant was discarded, and the pellet was resuspended in deionized water. The centrifugation and washing processes were repeated in triplicate. Subsequently, the purified Cu_2_O pellet was dispersed in ethanol for storage and use in subsequent experiments.

### Surface Modification of the Cu_2_O NPs with Serine

A 1 mL portion of 1 mM serine was thoroughly mixed with 4 mL of diluted phosphate-buffered saline (1× PBS) solution under vortex agitation. Then, 1 mL of Cu_2_O colloid at a 1000 ppm Cu concentration was added to the as-prepared serine solution and shaken for 10 min to form the serine-grafted Cu_2_O colloid. After the reaction, the colloidal solution was aliquoted and centrifuged at 7500 rpm for 5 min. Afterward, the supernatant was discarded, and the pellet was dispersed in fresh ethanol. The centrifugation and washing processes were repeated in triplicate. Finally, the purified serine-grafted Cu_2_O NPs were stored in ethanol for use in subsequent experiments.

### Deposition of Colloid on the Indium Tin Oxide Electrode

A 100 μL portion of serine-modified Cu_2_O NPs at a 1000 ppm Cu concentration was prepared. Then, the colloidal solution was homogeneously covered on the surface of ITO glass with a defined area (3 cm × 3 cm), followed by complete evaporation to form a uniform thin film of serine-grafted Cu_2_O NPs on the electrode. The NP-modified electrode was stored in a vacuum box to avoid the oxidation of Cu_2_O before the use of the experiments.

### Electrochemical Analysis

0.0104 g of β-HBA was dissolved in 10 mL of PBS to obtain a 10 mM β-HBA stock solution, and then, the working solutions with known concentrations were prepared after dilution with PBS. Then, 7 mL of the β-HBA working solution (10 mM) was added to an electrochemical reactor equipped with an NP-modified ITO working electrode, a Pt counter electrode, and a Ag/AgCl reference electrode for measurement in CV mode pulsed within a range from −0.5 to 0.8 V. For the β-HBA quantification measurement, 6 mL of PBS was added to the electrochemical reactor, and the measurement was performed by amperometry mode pulsed at −0.3, −0.1, and 0.1 V for 60 min to reach the current balance. Then, 1 mL of the β-HBA solution at known concentrations (0.01–20 mM) was added to the reactor to continuously record the current changes. β-HBA was replaced with glucose (4.4 mM) and dopamine (3 μM) under the same measurement conditions for specificity evaluation of the present detection method. All measurements were independently carried out in pentaplicate (*n* = 5). The equation of 3σ/slope was utilized to calculate the value of the detection limit, also known as the limit of detection.

### Characterizations

The morphology of the nanoparticle was observed by using transmission electron microscopy (TEM, Hitachi H-7500). An ultraviolet–visible (UV–vis) spectrometer (Analytik Jena Specord/200 Plus) was used to measure the optical characteristics of the NPs. Fourier transform infrared spectrometry (FTIR, Bruker Alpha1) was applied to obtain vibration spectra of NPs. The crystalline information of Cu_2_O NPs was identified by X-ray diffraction (XRD, Bruker, D8 ADVANCE). Dynamic light scattering (DLS, Otsuka Electronics, ELSZ-2000) analysis was used to assess the hydration diameter and zeta potential of NPs. The electrochemical measurements were performed by using a potentiostat (BioLogic, VSP-300) connected to three-electrode reactors.

## Results and Discussion

### Characterization of Cu_2_O-Serine NPs

The monosized Cu_2_O NPs were successfully synthesized by the redox reaction method referred to in the previous report.^10^ The observation in TEM imaging indicated the monodispersed Cu_2_O nanosphere with a size range of 225.2 ± 55.4 nm (Figure 1a). The nanoparticles exhibited a well-defined crystalline structure with characteristic peaks corresponding to the Cu_2_O composition, as evidenced by the XRD analysis (Figure 1b). Further surface modification of Cu_2_O NPs with serine by single chelated coordination between carboxylic acid and exposed Cu site was performed to form the serine-modified Cu_2_O NP, and this method is based on our previous study.^10^ The serine coordinated on the surface of NPs enables a specific conjugation with β-HBA, thus capturing β-HBA from the specimen during the subsequent electrochemical measurement. The UV–vis spectrum of Cu_2_O NPs reveals a strong absorbance at 455.5 nm, reflecting the innate feature of Cu_2_O in light absorption in the visible region (Figure 1c).^34^ Moreover, the UV–vis spectrum of serine shows a sharp absorption peak at 210 nm, and this peak can also be found on Cu_2_O-S NPs to evidence the successful surface modification of NP.^35^ There is no significantly different Cu_2_O-featured peaks in the spectra of Cu_2_O and Cu_2_O-S NPs, indicating the high stability of Cu_2_O during the serine coordination reaction. The result of the DLS analysis of NPs before and after serine grafting stated an increase in hydrodynamic diameter from 326.4 ± 13.6 to 505.7 ± 76.1 nm, implying the successful coordination of serine onto the surface of nanoparticles (Figure 1d). Zeta potential measurements demonstrated a notable change in surface charge from 17.13 to −6.63 mV after serine conjugation, attributed to the fact that the positively charged PVP surfactant was replaced with serine with oxygen-based hydroxy and carboxylic acid groups (Figure 1e). The open-circuit potential (OCP) measurements of Cu_2_O and Cu_2_O-S electrodes were performed to recheck the results of surfactant exchange. The OCP results indicated a positive potential of Cu_2_O and a negative potential of Cu_2_O-S at the beginning of the measurement, which shows a consistent tendency with zeta potential analysis of both electrodes (Figure S1). In addition, the vibration spectra obtained by FTIR analysis further support the evidence for successful modification of Cu_2_O NPs with serine (Figure 1f). The vibration peak at 618 cm^–1^, correlated to the Cu–O bonding, was detected from the NP. The sharp peak at 1626 cm^–1^ is attributed to the presence of PVP on the Cu_2_O NPs. The serine exists with four representative vibration peaks at 1038, 1427, 1507, and 1638 cm^–1^, correlated to C–O stretching, C–H bending, N–H bending, and C=O stretching, respectively. Notably, these characteristic peaks were also detected from Cu_2_O-S NPs, revealing the presence of serine on the surface of NPs.^36^ All of the characterization results indicate the good fabrication of Cu_2_O-S NPs, which was then deposited on the ITO electrode to form the NP-modified electrode for use in the subsequent electrochemical measurement sensing of β-HBA.

**Figure 1:**
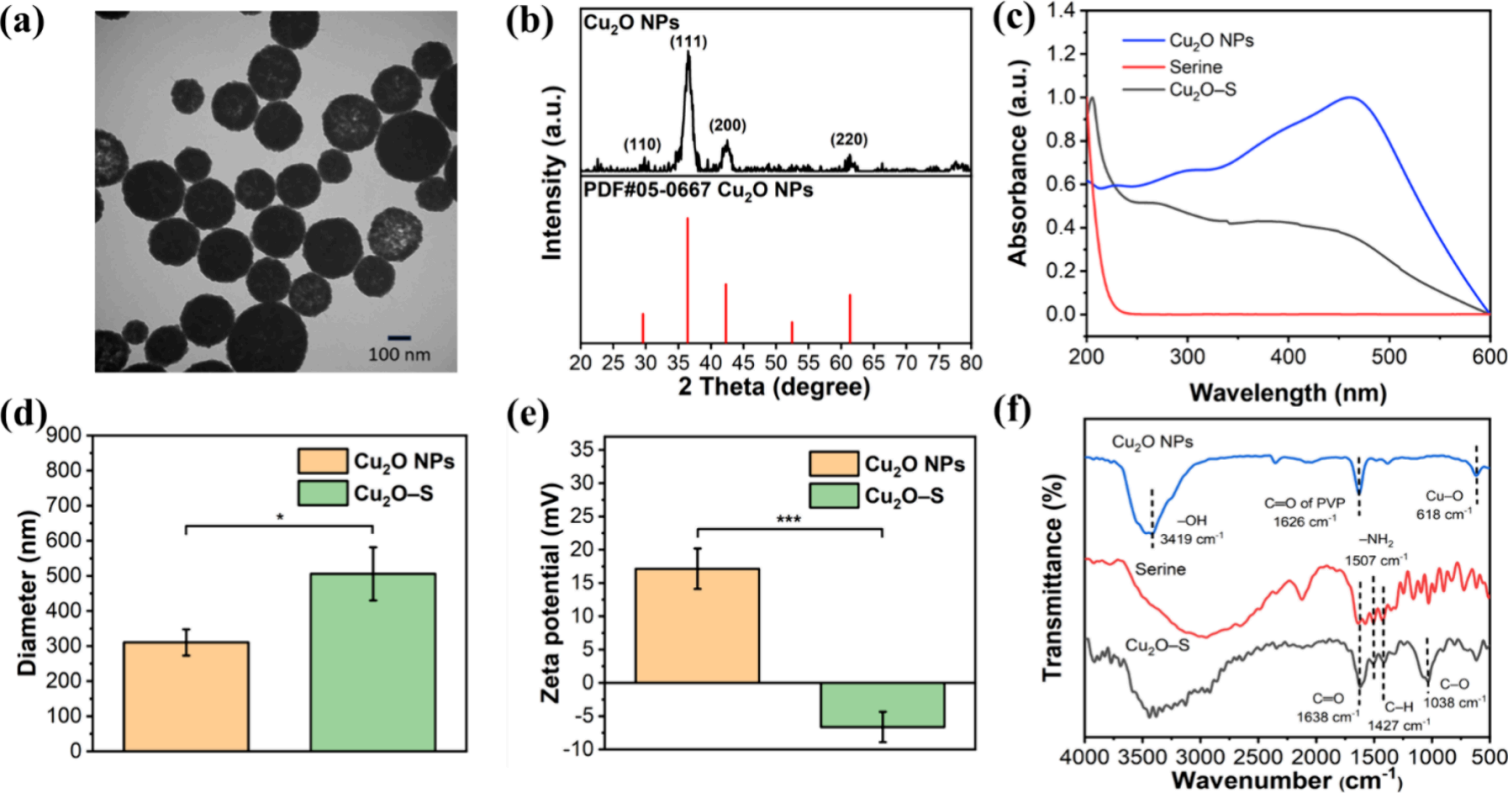
Characterization of the NPs. (a) TEM image of Cu_2_O NPs. (b) XRD profile of Cu_2_O NPs and standard diffraction peaks of copper­(I) oxide (PDF#05-0667). (c) UV–vis spectra of serine, Cu_2_O NPs, and Cu_2_O-S NPs. (d) Hydrodynamic diameter and (e) zeta potential of Cu_2_O NPs before and after amino acid grafting. (f) FTIR spectra of serine, Cu_2_O NPs, and Cu_2_O-S NPs (***p < 0.001; *p < 0.05).

### Electrochemical Detection of β-HBA Using NP-Modified Electrodes

In our concept, the Cu_2_O-S NPs on the ITO working electrode can rapidly capture the β-HBA dissolved in the medium through the alternative click conjugation between serine and β-HBA, thus measuring the β-HBA concentration-involved electrochemical signal changes (Figure 2a).^10^ Initially, CV analysis was performed to determine the electrochemical reactions within the system. The specific potential range of −0.5 to 0.8 V was considered and applied due to the unavoidable electrocatalytic water splitting involving the oxygen evolution reaction at 0.9 V and the hydrogen evolution reaction at −0.6 V.^37^ The CV result of the Cu_2_O electrode in the PBS system showed the two representative oxidative peaks at −0.09 and 0.4 V and one reductive peak at around −0.3 V, which, respectively, involved the oxidation of Cu^+^ and Cu_2_O to form the Cu^2+^ and CuO and reduction of Cu^+^ to form Cu^0^ (Figure 2b).^38^ Interestingly, the CV result of the Cu_2_O-S electrode reveals the peak disappearance at 0.4 V compared to that of the Cu_2_O electrode, implying that the serine modification on the surface of Cu_2_O can efficiently increase the stability of Cu_2_O to reduce the oxidation process (Figure 2c). The Cu_2_O-S electrode also shows equally matched oxidative and reductive peaks in multicyclic measurements. Dramatically, the significantly decreased peak of Cu^+^ oxidation at −0.09 V and enhanced intensity of Cu^+^ reduction at the potential of −0.3 V was found in the CV result of Cu_2_O-S + 1 mM β-HBA (Figure 2d).^38^ It seems to point out that the conjugation of serine and β-HBA on the surface of Cu_2_O can amplify the Cu reduction proportion. The CV profile of pure β-HBA and serine using an ITO electrode shows no significant peaks in the range of −0.5 to 0.8 V (Figures 2e and S2). A comparison of both results implies that electrocatalytic reactions in the Cu_2_O-S electrode system are related to Cu_2_O-S only, and no additional reaction of serine or β-HBA is observed.

**Figure 2:**
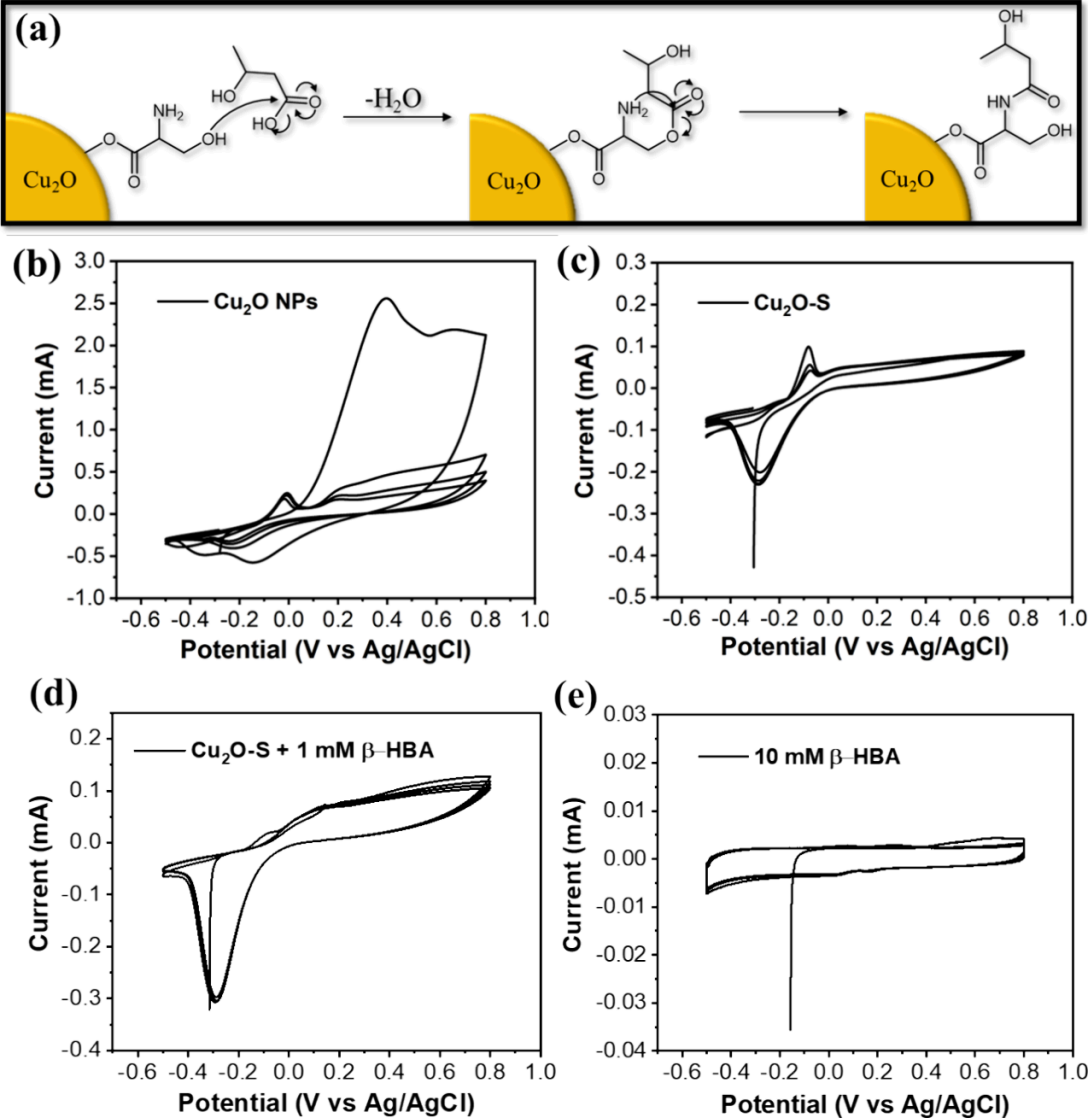
(a) Click chemistry-conjugable mechanism between serine and β-HBA. The cyclic voltammograms of (b) Cu_2_O-modified ITO electrode, (c) Cu_2_O-S-modified ITO electrode, (d) Cu_2_O-S-modified ITO electrode + β-HBA, and (e) bare ITO electrode + β-HBA.

Then, amperometry for the β-HBA quantitative experiment using the Cu_2_O-S electrode was conducted under a fixed voltage at −0.3, −0.1, and 0.1 V. For each measurement, β-HBA was injected into an electrochemical reactor after a 1 h prescan, which ensures that the system reaches a stable status before the β-HBA-involved reaction. Notably, the measurement at −0.1 V with increasing β-HBA amounts can increase the reductive current, revealing an excellent linear dynamic range between 0.01 and 20 mM β-HBA concentration and an applicable detection limit of 0.04 mM (Figure 3a,b). The average concentration of β-HBA in healthy human specimens is around 0.3 mM.^39^ A high accuracy and precision result was obtained to indicate the feasibility and reliability of the present approach for β-HBA analysis (Figure 3c).

**Figure 3:**
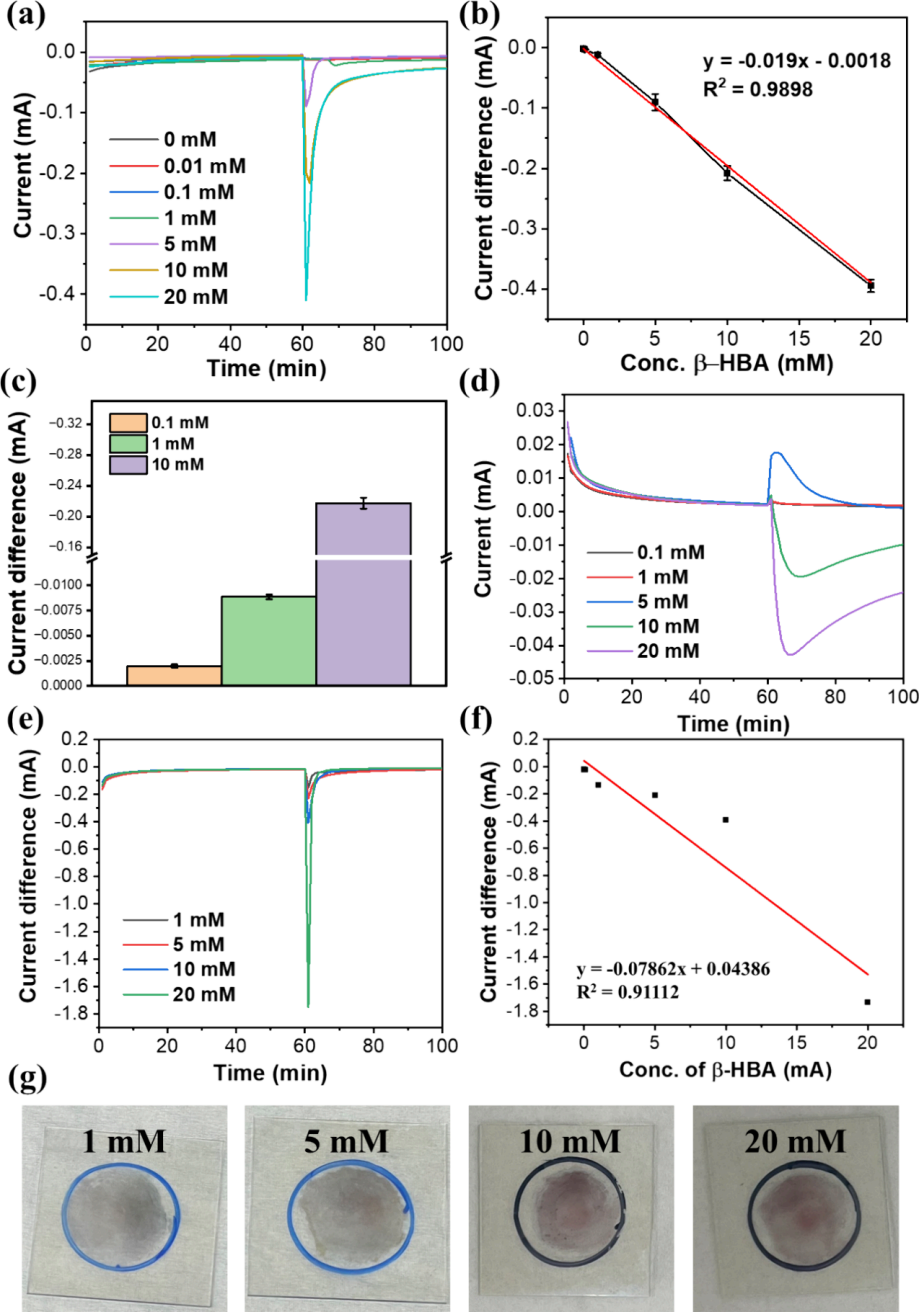
(a) Amperometry measurements at −0.1 V displaying current decreases after 60 min upon injecting different concentrations of β-HBA. (b) Linear regression analysis indicates a high correlation between the β-HBA concentration and current change (*R* ^2^ = 0.9898). (c) Current difference analysis of independent amperometry measurements of the Cu_2_O-S electrode with different concentrations of β-HBA at −0.1 V. All measurements were repeated in pentaplicate (*n* = 5). (d) Amperometry measurements at 0.1 V show different current responses after 60 min upon injecting different concentrations of β-HBA. (e) Amperometry measurement of the Cu_2_O-S electrode with different concentrations of β-HBA at −0.3 V. (f) Linear regression analysis of using the Cu_2_O-S electrode for β-HBA detection at −0.3 V (*R* ^2^ = 0.91112). (g) Photos of the electrode material after amperometry with different β-HBA concentrations.

It is worth noting that no significant reductive current was detected upon the 10 mM β-HBA measurement using a bare ITO electrode, implying that the primary electrochemical signal source has come from the electrolysis of Cu_2_O-S NPs (Figure S3). Without the serine grafting on the surface of Cu_2_O-S NPs, a relatively low reductive current was obtained, presenting that the alternative click conjugation of serine and β-HBA on the surface of the NP can efficiently facilitate the electrolysis process, thus increasing its sensitivity of β-HBA detection (Figure S4). On the other hand, no linear relationship between the β-HBA amount and current intensity was obtained under a positive potential at 0.1 V for measurement, which might be attributed to a complicated condition involving Cu^+^ and Cu_2_O oxidation reactions and the absence of Cu^+^ reduction (Figure 3d). Interestingly, the measurement at −0.3 V also indicated a relatively poor linear relationship and brown Cu^0^ formation, which might cause significant Cu^+^ reduction and the lack of Cu^+^ oxidation (Figure 3e–g). The β-HBA concentration-dependent increase of Cu^0^ formation was obtained, echoing the result of amperometry showing β-HBA concentration-dependent reductive current enhancement (Figure 3e,g).

### Artificial Sample Detection

In evaluating the specificity of the present method, common blood interferents, such as dopamine and glucose, were selected to test. The results showed negligible current differences with and without these interferents, and β-HBA can also be detected from a mixture, indicating satisfactory specificity of β-HBA detection using the Cu_2_O-S electrode (Figure 4a). An artificial blood sample was prepared by adding 5 mM β-HBA to mouse serum. Then, a standard amperometry measurement of β-HBA using the Cu_2_O-S electrode was performed, and the standard calibration curve was applied (Figures 3b and 4b). This preliminary result indicated that the sample contained 5.48 mM β-HBA to imply the good feasibility of the method presented here for biological sample analysis.

**Figure 4:**
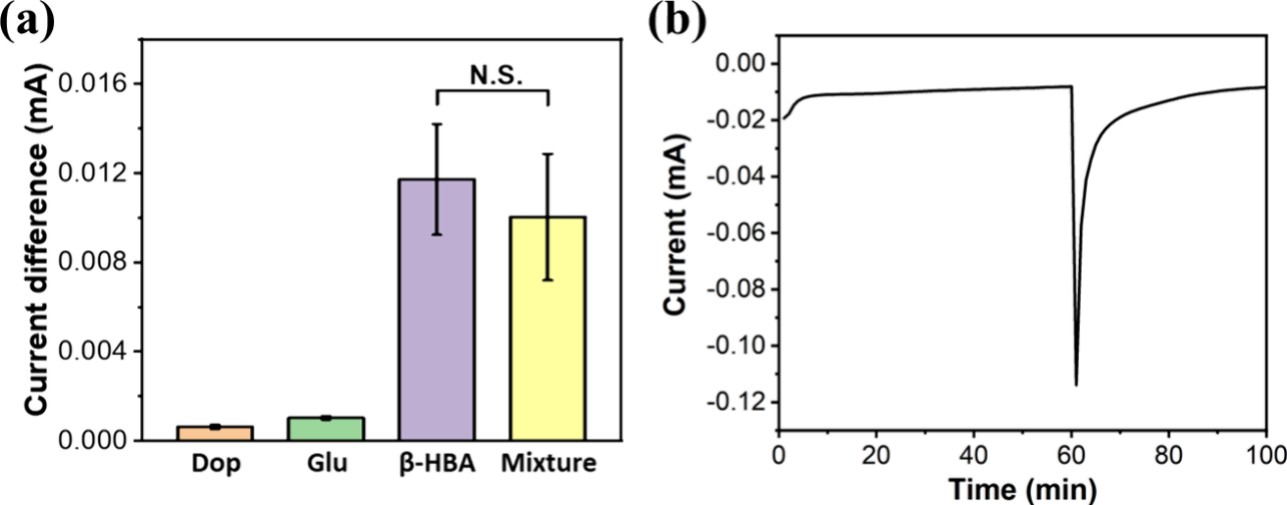
(a) Values of current change in the presence of common blood interferents at physiological concentrations: glucose concentration of 4.4 mM,^40,41^ and dopamine concentration of 3 μM, compared to β-HBA tested at 1 mM (N.S., no significance). (b) Amperometry measurement of the Cu_2_O-S electrode with an artificial sample containing 5 mM β-HBAs at −0.1 V.

### Surface Sensitization-Promoted Electrolysis

Different from the result of Cu^0^ formation upon the electrochemical measurement at −0.3 V, a noticeable yellow-to-blue color change of the Cu_2_O-S electrode before and after the electrochemical reaction at the potential of −0.1 V was observed (Figure 5a). This observation suggests that the Cu_2_O composition changes to form another new product during the electrolysis at −0.1 V, and the surface-conjugated β-HBA can promote this electrolysis. Under TEM observation, the newly resulting electrolytic product on the electrode shows a different morphology than the original Cu_2_O nanosphere (Figure 5b). Based on additional XRD analysis, the blue product belongs to the Cu_4_H­(PO_4_)_3_ composition and reveals poor crystallinity (Figure 5c). The UV–vis spectrum of the electrolytic product showed retained serine and lost Cu_2_O absorption signals, meaning the degradation of Cu_2_O composition. Moreover, a new peak appeared at 271 nm, which seemed to match with the ligand-to-metal charge transfer absorption of Cu_4_H­(PO_4_)_3_, fully supporting the fact of composition transformation from yellow copper­(I) oxide into blue copper­(II) phosphate (Figure 5d).^42^ Based on these findings, it is inferred that copper ions released from Cu_2_O degradation react with hydrogen phosphate and phosphate in the PBS electrolyte to form Cu_4_H­(PO_4_)_3_ during the electrolysis process (Scheme 1). Therefore, upon the electrochemical measurement pulsed at −0.1 V, Cu^+^ oxidation at −0.09 V leads to the formation of blue Cu_4_H­(PO_4_)_3_ crystals and Cu^+^ reduction at −0.3 V generates Cu^0^, which are two coexisting reactions during the Cu_2_O electrolysis. In addition, the critical β-HBA grafted on the surface of NPs might contribute to the sensitization of Cu^+^ reduction, resulting in the reductive current increase in their amperometry results (Figure 3a).

**Figure 5:**
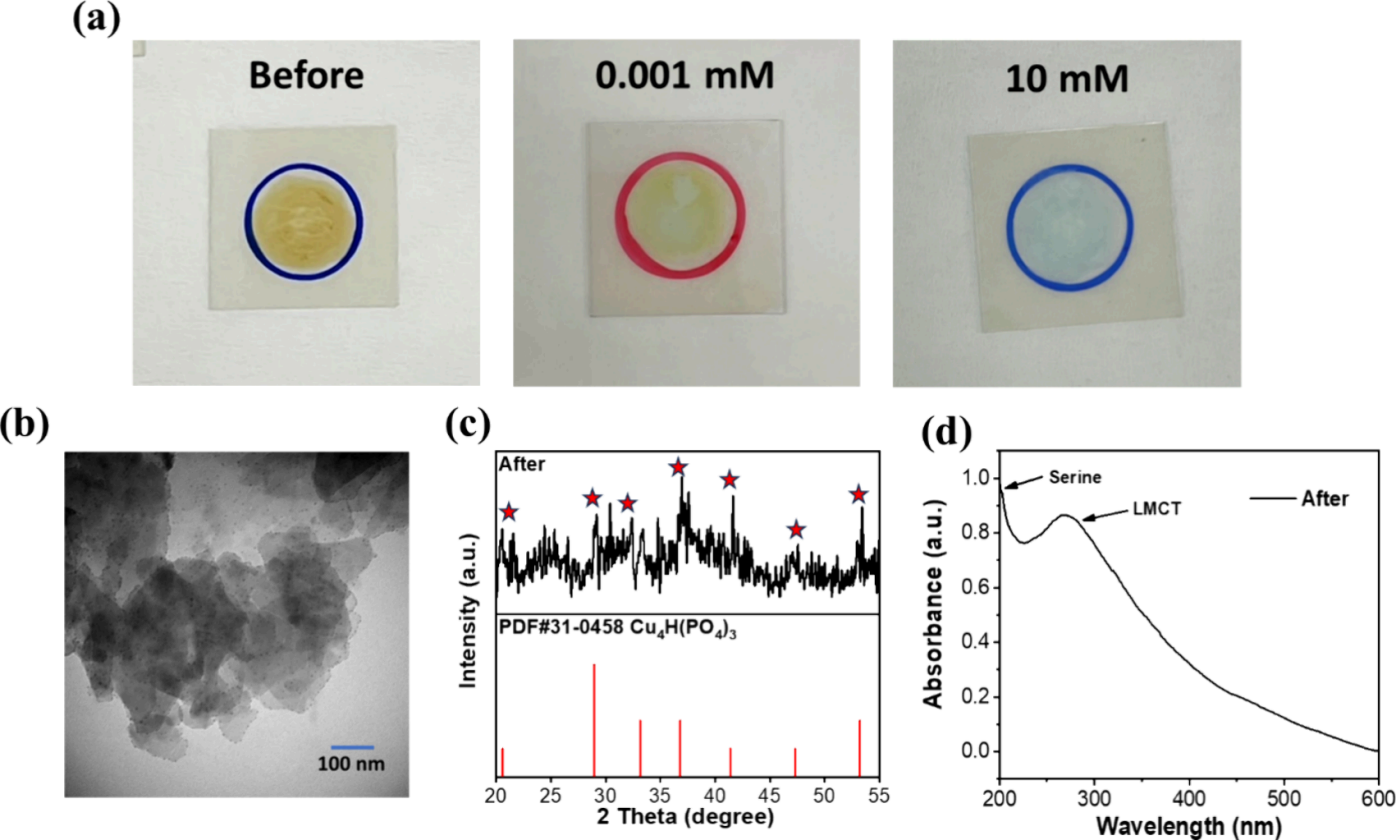
(a) Visible color change on the electrode material after amperometry, and the blue one is the group injected with 10 mM β-HBA. Characterization of the material after the electrochemical testing. (b) TEM image of the sheet-like shapes. (c) XRD spectroscopy matched the standard card (PDF#31-0458) for Cu_4_H­(PO_4_)_3_. (d) UV–vis spectroscopy of the resulting electrolytic materials.

## Conclusions

The serine-grafted Cu_2_O NP was successfully fabricated and applied to modify the ITO working electrode. The Cu_2_O-S NP enables conjugation with β-HBA through an alternative click chemical reaction, by which β-HBA in the specimen can be rapidly captured and deposited on the surface of the NP-modified electrode. Upon a pulse at −0.1 V, the β-HBA amount-dependent reductive current production was obtained, revealing an excellent linear dynamic range and an applicable detection limit. Based on additional observations of the composition changes before and after chronoamperometry, the critical role of Cu_2_O electrolysis was proposed. Overall, a combination of β-HBA-serine click conjugation and surface sensitization-promoted electrolysis reveals the potential to achieve specific and sensitive β-HBA detection, pointing out a new direction for developing small-molecule sensors.

## Supplementary Material



## Notes

Comparison of different strategies to detect β-HBA; open-circuit potential analysis; CV profiles; and amperometry results (PDF)
