# Supplementary material for: Serine-Grafted Cu2O Electrode Enabling Specific β‑Hydroxybutyrate Detection by Surface Sensitization-Promoted Electrolysis in Amperometry
Source: Langmuir. 2025 May 7;41(19):12022–9. doi: 10.1021/acs.langmuir.5c00591 (PMC12100702; doi:10.1021/acs.langmuir.5c00591)
Supplement: Supplementary file 1 [file la5c00591_si_001.pdf]

Supporting Information

Serine-Grafted Cu_2O Electrode Enabling Specific β-Hydroxybutyrate Detection by Surface Sensitization-Promoted Electrolysis in Amperometry

Ting-Chi Lo[1], Wen-Jyun Wang[1], Chih-Yen Chen[2,], Jui-Cheng Chang[3,*], and Wei-Peng Li[1,4,5,6,*]*

[1]Department of Medicinal and Applied Chemistry, Kaohsiung Medical University, Kaohsiung 807, Taiwan

[2]Department of Electrophysics, National Yang Ming Chiao Tung University, Hsinchu 300, Taiwan

[3]Department of Chemical Engineering, Chung Yuan Christian University, Taoyuan 320, Taiwan

[4]Department of Medical Research, Kaohsiung Medical University Hospital, Kaohsiung 807, Taiwan

[5]Drug Development and Value Creation Research Center, Kaohsiung Medical University, Kaohsiung 807, Taiwan

[6]Center of Applied Nanomedicine, National Cheng Kung University, Tainan 701, Taiwan

*Corresponding Email: chihyenchen@nycu.edu.tw; chang_juicheng@cycu.edu.tw; wpli@kmu.edu,tw

KEYWORDS: serine, β-hydroxybutyrate, nanoparticle, biosensor, amperometry

Contents

Table S1……………………………………………………………………………..…………… S3, 4

Figure S1……………………………………………………………………………………… S5

Figure S2……………………………………………………………………………………… S6

Figure S3……………………………………………………………………………………… S7

Figure S4……………………………………………………………………………………… S8

Method	Material	pH value	Linear range(mM)	R^2 value	Reference
**Fluorescent**	Fluorescent Probe	7.4	0~0.05	0.96	[6]
**Fluorescent**	Fluorescent Probe	7.4	0.003–0.06	0.9900	[7]
**Fluorescent**	NAD—QDs	7.4	0.008~10	0.9511	[8]
**CA**	2DPCH	7.0	1~10	0.995	[9]
**CA**	Au@Cu_2O—Serine NCs	4.5	0.001~10	0.985	[10]
**PEC**	β-HBDH—HRP/NPG/FTO	7.0	0.001~8	0.993	[11]
**EC**	SPCE/Au/TBO—Chit—CNTs	7.4	0.1~3	0.97	[12]
**EC**	$[Ru(bpy)_3]^{2+}$—GO/NAD/HBDH	7.4	0.2~2	0.9395	[13]
**EC**	Electrochemical Latent Redox Probe	8.0	0.01~0.6	0.989	[14]
**EC**	Ab@E-rGO/SPE	7.4	0.7~2	0.99	[15]
**EC**	Cu_2O—Serine NPs	7.4	0~20	0.9898	This work

Table S1. The comparison of different strategies to detect β-HBA. (NAD: Nicotinamide Adenine Dinucleotide, QDs: Quantum Dots, CA: Colorimetric Assay, 2DPCH: 2D Photonic Crystal Hydrogel, NCs: Nanocatalysts, PEC: Photoelectrochemical Reaction, β-HBDH: D-3-hydroxybutyrate Dehydrogenase, HRP: Horseradish Peroxidase, NPG: Nanoporous Gold, FTO: Fluorine-doped Tin Oxide**,** EC: Electrochemical Reaction, SPCE: Screen Printed Carbon Electrode, TBO: Toluidine Blue O, Chit: Chitosan, CNTs: Carbon Nanotubes, GO: Graphene Oxide, Ab: Anti-βHBA Antibodies, E-rGO: Electrochemically Reduced Graphene Oxide, SPE: Screen Printed Electrode, NPs: Nanoparticles)

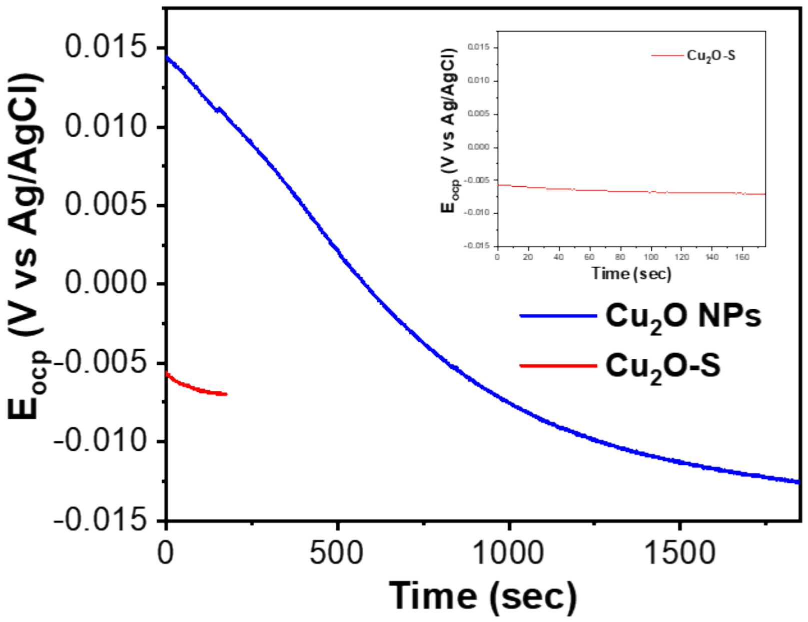

Figure S1. The open circuit potential measurements of Cu_2O-modified ITO electrode and Cu_2O-S-modified ITO electrode. The measurements were automatically terminated when the potential achieved a stable state. The insert is the magnified curve of the Cu_2O-S-modified ITO electrode. The drastic potential change of Cu_2O might be attributed to salt adsorption. The potential of Cu_2O-S is relatively stable during OCP measurement.

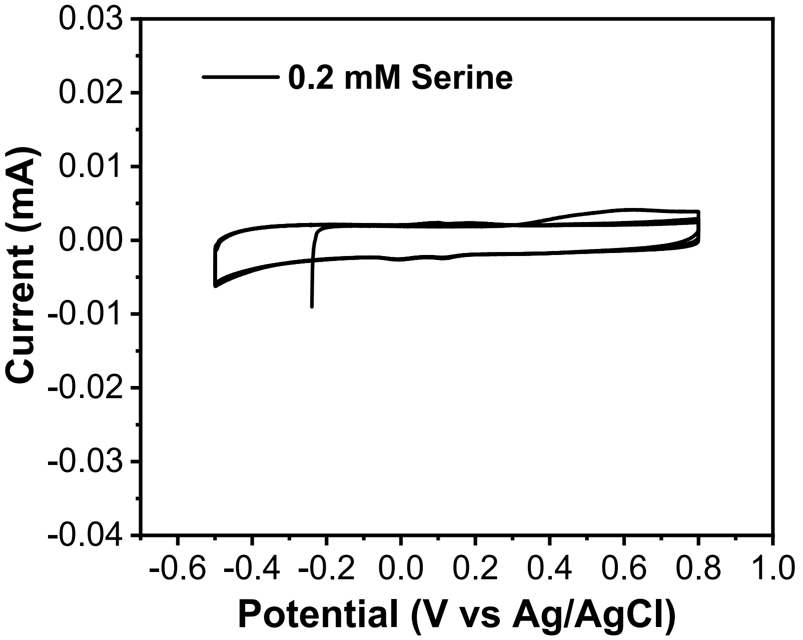

Figure S2. The cyclic voltammograms of bare ITO electrode + serine. The PBS buffer was used for the above measurement.

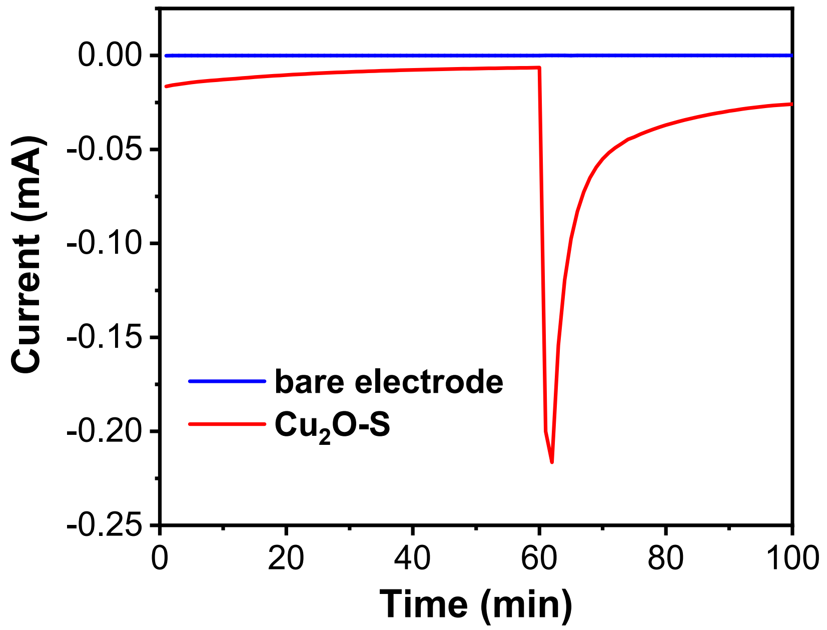

Figure S3. Comparison of amperometry measurements with and without Cu_2O-S modification on the electrode after injecting 10 mM β-HBA for 60 minutes.

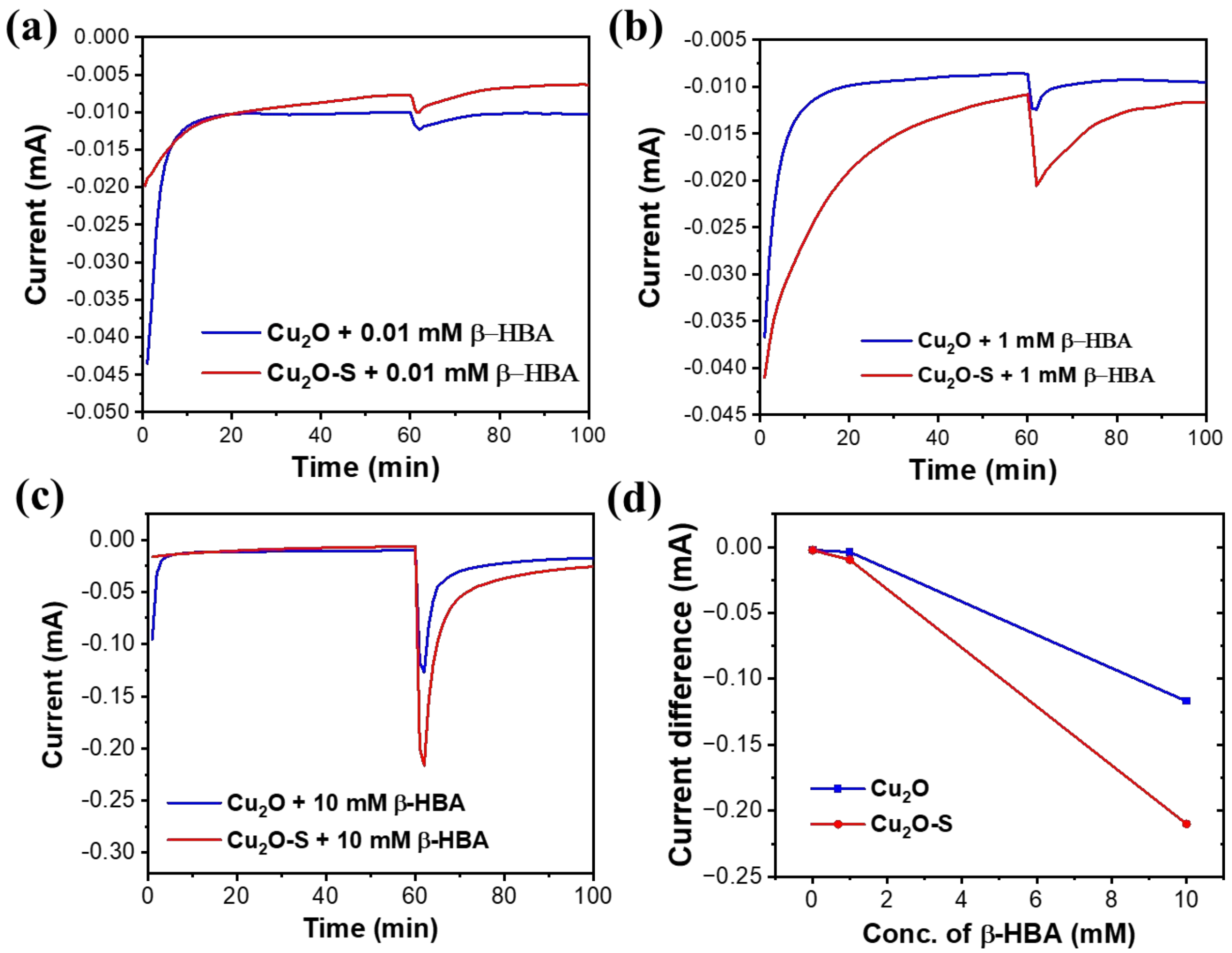

Figure S4. Comparison of amperometry measurements for Cu_2O with and without connecting serine. Amperometry profiles of Cu_2O and Cu_2O-S electrode with (a) 0.01, (b) 1, and (c) 10 mM of β-HBAs at the potential of -0.1 V. (d) Linear analysis of using Cu_2O and Cu_2O-S electrodes for β-HBA detection at -0.1 V.
